# The Impact of HIF1α on the *Per2* Circadian Rhythm in Renal Cancer Cell Lines

**DOI:** 10.1371/journal.pone.0109693

**Published:** 2014-10-21

**Authors:** Takashi Okabe, Megumi Kumagai, Yoshihiro Nakajima, Suguru Shirotake, Kiichiro Kodaira, Masafumi Oyama, Munehisa Ueno, Masaaki Ikeda

**Affiliations:** 1 Department of Uro-oncology, Saitama Medical University International Medical Center, Saitama, Japan; 2 Department of Physiology, Saitama Medical University, Saitama, Japan; 3 Molecular Clock Project, Project Research Division, Research Center for Genomic Medicine, Saitama Medical University, Saitama, Japan; 4 Health Research Institute, National Institute of Advanced Industrial Science and Technology (AIST), Kagawa, Japan; University of Texas Southwestern Medical Center, United States of America

## Abstract

In mammals, the circadian rhythm central generator consists of interactions among clock genes, including *Per1/2/3*, *Cry1/2*, *Bmal1*, and *Clock*. Circadian rhythm disruption may lead to increased risk of cancer in humans, and deregulation of clock genes has been implicated in many types of cancers. Among these genes, *Per2* is reported to have tumor suppressor properties, but little is known about the correlation between *Per2* and HIF, which is the main target of renal cell carcinoma (RCC) therapy. In this study, the rhythmic expression of the *Per2* gene was not detectable in renal cancer cell lines, with the exception of Caki-2 cells. In Caki-2 cells, HIF1α increased the amplitude of *Per2* oscillation by directly binding to the HIF-binding site located on the *Per2* promoter. These results indicate that HIF1α may enhance the amplitude of the *Per2* circadian rhythm.

## Introduction

Renal cell carcinoma (RCC) is the most common malignancy of the adult kidney, which accounts for approximately 2% of cancers worldwide [1]. A somatic mutation of the Von Hippel–Lindau (*VHL*) gene is the most frequent genetic change observed in RCC [2], and recent efforts have targeted the VHL–hypoxia inducible factor (HIF)-mediated hypoxia-induced gene pathway for RCC therapy [3]. HIFs are heterodimeric transcription factors with two structurally related subunits: an oxygen-sensitive HIFα subunit and a constitutively expressed HIFß or aryl hydrocarbon receptor nuclear translocator (ARNT) subunit [4]. In normoxia, HIFα molecules are subjected to a regulatory process involving enzymatic hydroxylation of conserved prolyl and asparaginyl residues, leading to rapid VHL protein-mediated ubiquitination and proteasomal degradation [5]. Hypoxia or mutations in the *VHL* gene inactivate this pathway. Increased HIFα activity upregulates genes involved in many aspects of cancer progression, including metabolic adaptation, apoptotic resistance, and angiogenesis [3]. In RCC, intense tumor vascular networks can be attributed to the inappropriate accumulation of HIFα leading to angiogenic gene induction. Vascular endothelial growth factor (VEGF) is one of the most potent pro-angiogenic factors, whose expression is transactivated by HIF1α/ARNT through binding to the hypoxia-response element (HRE) in the *Vegf* promoter [6], [7]. Increased expression of VEGF is also associated with malignant progression and a poor treatment outcome [8]. Therefore, suppressing the HIF-mediated gene pathway may be an important therapeutic strategy for the treatment of RCC [3].

Many physiological, biochemical, and behavioral processes are under circadian regulation, which is generated by an internal time-keeping mechanism referred to as the biological clock in almost all organisms from bacteria to mammals [9], [10]. Circadian rhythms are controlled by genetically determined networks of transcription–translation feedback loops involving clock genes, including *Per1/2/3*, *Cry 1/2*, *Bmal1*, and *Clock* [11]. A common theme underlying circadian rhythmicity is that oscillations of clock gene transcripts are the consequence of intracellular transcriptional–translational feedback loops. For example, in mammals, the transcription factors CLOCK and BMAL1 heterodimerize and activate the expression of three *Per* genes and two *Cry* genes by binding to E-box elements in their promoters. The protein products of these genes multimerize and translocate to the nucleus, where PER and CRY proteins repress the transcriptional activity of the CLOCK–BMAL1 dimer [12], [13].

Among these clock genes, *Per2* is responsible for setting the period of oscillation [14]. Furthermore, *Per2* has tumor-suppressor properties and is often mutated or downregulated in human breast cancers [15], [16]. In renal cancer, altered expression of the *Per2* gene is reportedly involved in disease onset and progression, but the molecular mechanism responsible remains unclear [17].

In this study, we measured the levels of *Per2* promoter activity and mRNA in eight renal cancer cell lines after dexamethasone treatment. The *Per2* promoter activity and mRNA level oscillated over an approximately 24-h cycle in Caki-2 cells, which contain BMAL1, CLOCK, and HIF1α proteins. We also found that HIF1α increased the amplitude of oscillation by directly binding to the HRE-like element located on the *Per2* promoter. These results show that HIF1α may affect the amplitude of *Per2* circadian rhythms in renal cancer cell lines.

## Materials and Methods

### Cells and cell cultures, chemicals, and enzymes

Established human RCC cell lines (A704, ACHN, 786-O, A498, 769-P, and Caki-2) were obtained from the American Type Culture Collection (ATCC; Manassas, VA, USA). RCC4+vector alone and RCC4+VHL were obtained from Sigma (St. Louis, MO, USA). These renal cell lines were maintained in Roswell Park Memorial Institute (RPMI)-1640 medium (Kojin Bio, Tokyo, Japan) supplemented with 10% fetal bovine serum (FBS; Life Technologies, Carlsbad, CA, USA), 24 U/mL penicillin, and 25 µg/mL streptomycin (Gibco, Grand Island, NY, USA) in a standard humidified incubator at 37°C in an atmosphere of 5% CO_2_. We also used the mouse fibroblast NIH3T3 and human osteosarcoma U2OS cell models of the autonomous circadian clock [18], [19]. These cell lines were also obtained from ATCC, and were maintained in Dulbecco's modified Eagle's medium (DMEM), supplemented with 10% FBS, penicillin (24 U/mL), and streptomycin (25 µg/mL). Chrysin was purchased from Sigma, and its purity exceeded 96%. A stock solution of chrysin was prepared in dimethyl sulfoxide (DMSO). Chrysin was dissolved in DMSO at three different concentrations (1, 10, and 100 mM) and added each 2 µL to 2 mL culture media (final concentration; 1, 10, 100 µM). Cells were treated with culture media containing 1, 10, 100 µM chrysin or same concentration of DMSO as control for 2 hours.

### Plasmid construction

To construct reporter vectors carrying the m*Per2* promoter, the m*Per2* promoter fragment (−279 to +112 bp, where +1 indicates the putative transcription start site) was polymerase chain reaction (PCR)-amplified from the C57BL/6J mouse genome, and cloned into the NheI/XhoI site of pGL3 Basic (Promega, Madison, WI, USA). Firefly luciferase (FLuc) was replaced with the *Nco*I and *Xba*I fragment of pSV40-dFLuc, resulting in m*Per2*-dFLuc. The HRE-mutant m*Per2* promoter reporter was generated with inverse PCR using a KOD-Plus-Mutagenesis Kit (Toyobo, Osaka, Japan).

### Real-time reporting of circadian-regulated gene expression using luciferase bioluminescence

All cells were seeded (5×10^4^ per dish) in a 35-mm dish 2 days before transfection, and the reporter plasmid was transfected using Lipofectamine 2000 (Invitrogen, Carlsbad, CA, USA) according to the manufacturer's instructions. The appropriate amount of reporter plasmid for each cell line was determined according to differences in transfection efficiency among the cell lines. One day after transfection, cells were treated with 100 nM dexamethasone (Nakalai Tesque, Kyoto, Japan) for 2 h, and the medium was replaced with medium in the absence of phenol red supplemented with 10% FBS and 100 µM D-luciferin (Toyobo). Bioluminescence was measured at 37°C under a 5% CO_2_ atmosphere and integrated for 1 min at intervals of 10 min using a dish-type luminometer, AB-2550 Kronos Dio (ATTO, Tokyo, Japan) [20], [21]. Bioluminescence activity was expressed as relative light units (RLUs). Each experiment was repeated at least four times. The cells were cultured in the luminometer for at least 4 days while the instrument counted their bioluminescence. The obtained crude data (10-min bins) were smoothed by a 10-point moving average method and detrended by subtracting a 12-h moving average from the smoothed data [21].

### Analysis of circadian rhythms using bioluminescence

To test the significance of the circadian rhythmicity and to calculate circadian parameters (i.e., period, amplitude, and acrophase), we performed computerized data analysis in the Cosinor software downloaded from the Circadian Rhythm Laboratory (Walterboro, SC, USA) software home page (http://www.circadian.org/software.html) [22], [23]. Circadian parameters were calculated using data from 1–5 days after dexamethasone treatment.

### Automated image capture and analysis

NIH3T3 cells were seeded (5×10^4^ per well) on 6-well plates 1 day before transfection, and the expression plasmid was transfected using Lipofectamine 2000 (Invitrogen) according to the manufacturer's instructions. One day after transfection, cells were treated with 100 nM dexamethasone (Nakalai Tesque) for 2 h, and the medium was replaced with medium in the absence of phenol red supplemented with 10% FBS. Cells were stained with 0.1 µg/mL Hoechst 33342 (Invitrogen) for 1 hour and analyzed using ArrayScan XTI (Thermo Scientific, Waltham, MA, USA).

### Quantification of mRNA by real-time RT-PCR

All cells were harvested at 4-h intervals from six plates at each time point beginning 24 h after treatment with dexamethasone. Total RNA from these cells was extracted using ISOGEN (Nippon Gene, Tokyo, Japan) and reverse transcribed. *Per2* and *Gapdh* transcripts were quantified using an ABI Prism 7300 (Applied Biosystems, Foster City, CA, USA). PCR was performed using the One Step SYBR PrimeScript RT-PCR Kit (Takara Bio, Kyoto, Japan) with the following thermal cycling parameters: 94°C for 5 min followed by 40 cycles at 94°C for 20 s and 62°C for 1 min. The *Gapdh* transcript was used to normalize the expression of each transcript. Circadian rhythmicity significance was analyzed using the Cosinor software (Circadian Rhythm Laboratory) [22], [23]. Primers for each gene were designed based on the information available from the National Center for Biotechnology Information (NCBI). The PCR primer sequences were as follows:

*Per2* (GenBank accession no., NM_022817; amplicon, 85 bp): sense primer 5′-CACACACAGAAGGAGGAGCA-3′ and antisense primer 5′-AGTAATGGCAGTGGGACTGG-3′.

*Gapdh* (GenBank accession no., M33197; amplicon, 185 bp): sense primer 5′-GAGTCAACGGATTTGGTCGT-3′ and antisense primer 5′-GACAAGCTTCCCGTTCTCAG-3′.

### Luciferase assay

Transfected NIH3T3 cells were used for luciferase assays. One day before transfection, cells were seeded (5×10^4^ per well) on 24-well plates containing DMEM supplemented with 10% FBS, penicillin (24 U/mL), and streptomycin (25 µg/mL). Cells were transfected using Lipofectamine 2000 (Invitrogen). For each sample, transfected DNA was added to each well. Twenty-four hours after transfection, cells were washed in phosphate-buffered saline (PBS) and disrupted with 100 µL of passive lysis buffer (Promega). Luciferase activity was determined using a Dual-Luciferase Reporter Assay System (Promega) and an Ascent FS II luminometer (Thermo Scientific).

### Western blotting

All cells were synchronized by 100 nM dexamethasone treatment for 2 h. Then, the medium was replaced with fresh medium. After 24-h incubation, these cells were lysed in Cell Lytic-MT (Sigma). The cell lysates were centrifuged at 15,000 rpm at 4°C for 10 min. The supernatants were stored as whole cell extracts at −80°C until use. For Western blotting, 20- µg protein were resolved on 7.5% sodium dodecyl sulfate polyacrylamide (SDS-PAA) gels and transferred onto a nitrocellulose membrane (Bio-Rad, Hercules, CA, USA). The membranes were blocked with Tris-buffered saline (TBS)-Tween containing 5% non-fat dried milk. Proteins were detected using antibodies against HIF1α (dilution, 1: 500; BD Transduction Laboratories, Franklin Lakes, NJ, USA), PER2 (dilution, 1∶1000; Santa Cruz Biotechnology, Santa Cruz, CA, USA), CRY1 (dilution, 1∶2000; Santa Cruz Biotechnology), CLOCK (dilution, 1∶1000; Thermo Scientific), GAPDH (dilution, 1∶10000; Sigma), and BMAL1 (dilution, 1∶100; mouse monoclonal antibody generated in our lab). We performed four replicate Western blots; a representative blot is shown.

### ChIP assay

ChIP experiments were performed using a commercially available kit according to the manufacturer's instructions (Magna-ChIP; Millipore, Bedford, MA, USA). Briefly, Caki-2 cells were plated in 100-mm diameter dishes (5×10^4^ cells per dish); after 24 h, cells were incubated with formaldehyde (final concentration, 1%) for 10 min at 37°C to cross-link proteins to DNA. Unreacted formaldehyde was quenched with 1 mL of 10× glycine. The plate was washed twice with ice-cold PBS, and the pellets were harvested in 1 mL PBS with protease inhibitor cocktail and pooled together in a 1.5-mL tube. The cross-linked chromatin was sheared by sonication 20 times for 1 min each time with 1 min of cooling on ice between pulses using a Branson 2510 Ultrasonic Cleaner (Branson, Danbury, CT, USA). Immunoprecipitation (IP) was performed with 5 µg of either anti-HIF1α (dilution, 1: 500; Novus Biologicals Inc., Littleton, CO, USA) or anti-IgG antibody (Millipore) as a negative control. Washes and elution of the IP DNA were performed according to the Magna-ChIP protocol (Millipore). Ten percent (10%) of the original sheared chromatin DNA was similarly reverse cross-linked and purified, and the recovered DNA was used as an input control. PCR was performed with specific primers flanking the HRE-like sequence within the promoter region of the human *Per2* gene (−476 to −284 bp, sense: 5′- ACGCCGGAAGTGGATGAGAC -3′ and antisense: 5′- CGACTCCGTCTCATCTGCATACAT -3′) with the following thermal cycling parameters: 94°C for 3 min, followed by 40 cycles at 94°C for 20 s, annealing at 59°C for 30 s, and extension at 72°C for 30 s.

### Statistical analysis

Each experiment was repeated at least four times. Data are expressed as means ± standard errors. To evaluate the significance of differences, Student's *t*-test was performed. We used a one-way analysis of variance (ANOVA) for comparisons among the drug concentration groups, followed by application of Tukey's post hoc tests. For all analyses, the significance level was set at *P*<0.05. The Cosinor software (Circadian Rhythm Laboratory) [22], [23] was used to analyze circadian rhythmicity.

## Results

### Circadian expression of the *Per2* gene in renal cancer cell lines

**Figure 1:**
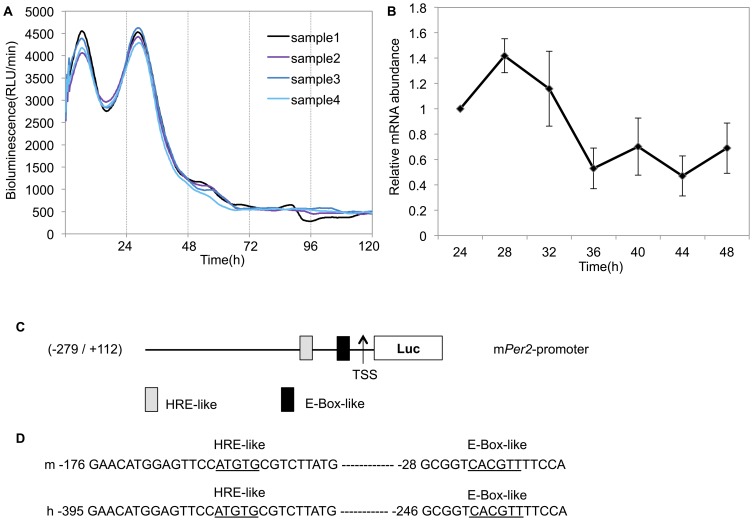
Rhythmic expression of *Per2* in Caki-2 cells. (**A**) All renal cancer cell lines were transfected with the Per2 promoter reporter (2 µg) and the bioluminescence was then measured using a real-time monitoring assay. Real-time monitoring of luciferase activity of the *Per2* promoter showed that activity oscillated over an approximately 24-h cycle. The luciferase activities of four replicate samples are shown. These cultures showed significant circadian rhythms (Table 1). (**B**) mRNA levels of *Per2* were determined by real-time PCR for six plates at each time point. Total RNA was extracted every 4 h, beginning 24 h after treatment with dexamethasone for one 24-h cycle, and *Per2* transcripts were quantified. Error bars indicate the standard errors of the mean values (*n* = 6). The data from a single 24 hours after dexamethasone treatment were analyzed using the Cosinor software for rhythmicity (Table 1). (**C**) The structure of the *Per2* promoter and an analysis of the potential transcription factor-binding motifs in this region. The 2,994-bp region contains one E-box-like sequence (CACGTT) and one HRE-like sequence (ATGTG), similar to the consensus HRE sequence (ACGTG) located upstream of the transcription start site (TSS). (**D**) Sequence comparisons: upper line, mouse sequence; lower line, human sequence. The nucleotide sequence of potential transcription factor-binding motifs for E-box-like sequence and HRE-like sequence are 100% conserved between mouse and human.

To explore the transcriptional oscillation of *Per2*, all cell lines were transfected with a luciferase reporter gene driven by the *Per2* promoter, and a real-time monitoring assay was performed using Kronos Dio (AB-2550; ATTO). A luciferase-bound promoter in Caki-2 cells displayed circadian rhythms after 2 h dexamethasone treatment (Fig. 1A, Table 1), but rhythmicity was not detected in the other cell lines (Fig. S1). Each experiment was repeated four times and these results were consistent. 24 h after dexamethasone treatment, *Per2* mRNA levels had a circadian rhythm in Caki-2 cells (Fig. 1B, Table 1). These results showed that the circadian rhythmicity of the *Per2* gene was not detectable in renal cancer cell lines, excluding Caki-2 cells.

**Table:** **Table 1.** Circadian parameters of *Per2* promoter activities and mRNA in Caki-2 cells.

	period (h)	amplitude	acrophase (h)	P value
Promoter activitiy	24.18±0.05	442.24±16.72	6.17±0.11	<0.000001
mRNA	N/A	0.4223	4.95	<0.05

Promoter activity and mRNA levels of *Per2* showed significant circadian rhythms (*p*<0.000001, *p*<0.05, by Cosinor).

### Analysis of the *Per2* promoter region

A previous study demonstrated that an E-box-like sequence (CACGTT) and its downstream region are essential for transcriptional oscillation of *Per2*, a crucial component of molecular clocks [24]. We focused on this E-box-like region and HRE. The transcription factor-binding motifs located on the *Per2* promoter in mice and humans were analyzed using MatInspector software (Genomatix, Munich, Germany). Sequence analysis of the *Per2* promoter region revealed high homology between mice and humans. Sequence analysis also revealed one E-box-like sequence (CACGTT) and one HRE-like sequence (ATGTG), similar to the consensus HRE sequence (ACGTG) [25] located upstream of the transcription start site (TSS) (Fig. 1C). These sequences were 100% conserved between mice and humans (Fig. 1D). A real-time monitoring assay (Fig. 1A) indicated that the promoter region we cloned is sufficient to produce circadian transcriptional oscillation in human cell lines.

### Expression of clock genes in renal cancer cell lines

**Figure 2:**
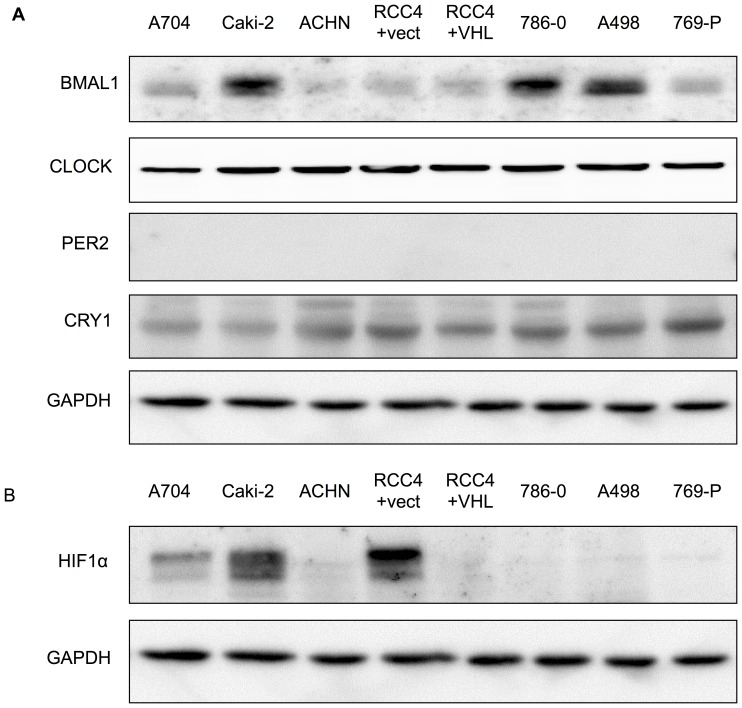
Western blot analysis of the indicated proteins. All cell lines were lysed and harvested 24 h after synchronization by 2-h dexamethasone treatment. We performed four replicate Western blots; a representative blot is shown. (**A**) Western blots of renal cancer whole-cell extracts (20 µg) with BMAL1, CLOCK, PER2, CRY1 and GAPDH antibodies are shown. Full-length blots of PER2 and BMAL1, in addition to a positive control, are presented in Figure S2, 3. (**B**) Western blots of renal cancer whole-cell extracts (20 µg) with HIF1α and GAPDH antibodies are shown.

To examine the difference between Caki-2 and other cell lines, we examined the expression of BMAL1, CLOCK, PER2, and CRY1 proteins. Caki-2, 786-O, and A498 cells expressed BMAL1 protein. All renal cancer cell lines expressed CLOCK and CRY1 protein, but did not express PER2 protein (Fig. 2A). Full-length blots of PER2 and BMAL1, in addition to a positive control, are presented in Figure S2, S3.

### Expression of HIFα protein under normoxic conditions in renal cancer cell lines

Since the HIF1α protein may be generally overexpressed in RCC, we also examined the expression of HIF1α protein. In Caki-2 and RCC4+vector alone, HIF1α protein was overexpressed (Fig. 2B). Considering the results that the *Per2* circadian rhythm was shown only in Caki-2 cells, which contained BMAL1, CLOCK, and HIF1α protein, it is possible that HIF1α is related to the *Per2* circadian rhythm in renal cancer cell lines.

### The impact of HIF1α on *Per2* transcriptional activity

**Figure 3:**
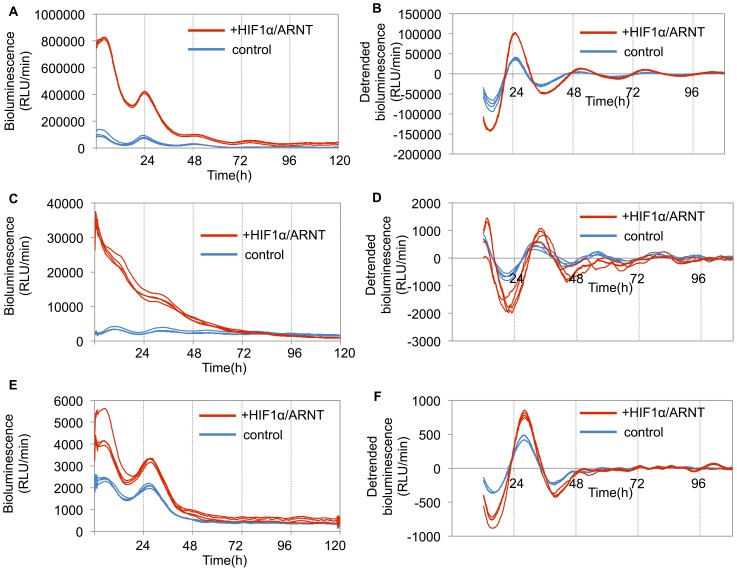
The impact of HIF1α/ARNT on *Per2* transcriptional activity. (**A**) NIH3T3 cells were co-transfected with the *Per2* promoter reporter (400 ng) and the indicated expression plasmids (300 ng) for HIF1α/ARNT or empty vector pcDNA3 (600 ng) as a control. Bioluminescence was then measured using a real-time monitoring assay. Control, transfected with empty vector pcDNA3 (uncloned-vector control); + HIF1α/ARNT, transfected with the expression plasmids. Luciferase activities of four replicate samples are shown. (**B**) Detrended bioluminescence is shown. Period, amplitude, and acrophase of the oscillations were measured on days 2 to 5 using the Cosinor software (Circadian Rhythm Laboratory). Amplitude significantly increased (mean ± SEM, *n* = 4) compared to the control (*p*<0.01, Student's *t-*test). See Table 2. (**C**) U2OS cells were co-transfected with the *Per2* promoter reporter (400 ng) and the indicated expression plasmids (300 ng) for HIF1α/ARNT or empty vector pcDNA3 (600 ng) as a control. Bioluminescence was then measured using a real-time monitoring assay. Control, transfected with empty vector pcDNA3 (uncloned-vector control); + HIF1α/ARNT, transfected with the expression plasmids. Luciferase activities of four replicate samples are shown. (**D**) Detrended bioluminescence is shown. Period, amplitude, and acrophase of the oscillations were measured on days 2 to 5 using the Cosinor software (Circadian Rhythm Laboratory). Amplitude significantly increased (mean ± SEM, *n* = 4) compared to the control (*p*<0.01, Student's *t-*test). See Table 2. (**E**) Caki-2 cells were co-transfected with the *Per2* promoter reporter (2 µg) and the indicated expression plasmids (1.5 µg) for HIF1α/ARNT or empty vector pcDNA3 (3 µg) as a control. The bioluminescence was then measured using a real-time monitoring assay. Control, transfected with empty vector pcDNA3 (uncloned-vector control); + HIF1α/ARNT, transfected with the expression plasmids. The luciferase activities of four replicate samples are shown. (**F**) Detrended bioluminescence is shown. Period, amplitude, and acrophase of the oscillations were measured from days 2 to 5 using the Cosinor software (Circadian Rhythm Laboratory). Amplitude significantly increased (mean ± SEM, *n* = 4) compared to the control (*p*<0.01, Student's *t*-test). See Table 2.

To examine the impact of HIF1α on *Per2* transcriptional activity, NIH3T3 and U2OS cells were transfected with a luciferase reporter gene driven by the *Per2* promoter and co-transfected with *Hif1α* and *Arnt* expression vector. Co-transfection with HIF1α/ARNT increased the amplitude of oscillation and had no influence on the period or acrophase of oscillation in these cell lines (Fig. 3A–D, Table 2). The same results were observed in Caki-2 cells (Fig. 3E, F, Table 2).

**Table:** **Table 2.** Circadian parameters of *Per2* promoter activities based on four days of data.

		period	amplitude	acrophase	P value
Caki-2	control	24.18±0.025	191.16±5.11	5.18±0.06	<0.0001
	+HIF1α/ARNT	24.18±0.025	325.97±2.91**	5.33±0.04	<0.0001
NIH3T3	control	24.43±0.05	7930.86±442.12	2.90±0.05	<0.000001
	+HIF1α/ARNT	24.23±0.08	41876.88±33.40**	3.11±0.07	<0.000001
U2OS	control	23.83±0.13	220.63±31.95	7.52±0.76	<0.000001
	+HIF1α/ARNT	24.05±0.02	1121.36±58.72**	6.02±0.09	<0.000001

Period, amplitude, and acrophase of the oscillations were measured on days 2 to 5 using the Cosinor software (Circadian Rhythm Laboratory). Amplitude significantly increased (mean ± SEM, *n* = 4) compared to the control (** *p*<0.01, Student's *t-*test).

### HIF1α has no effect on number of NIH3T3 cells

**Figure 4:**
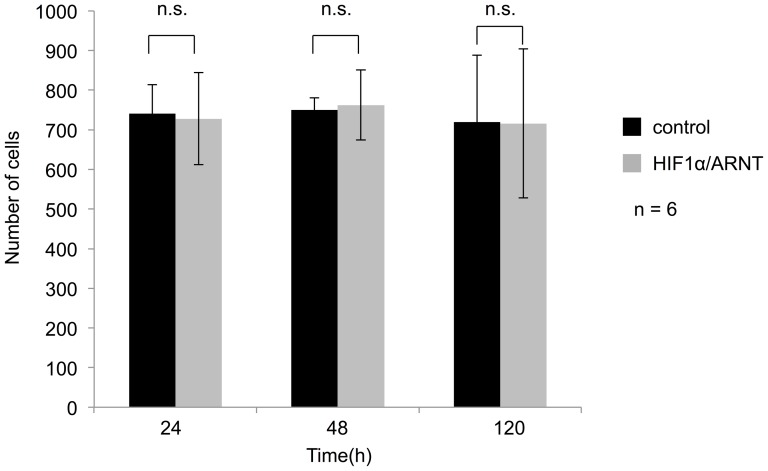
The effects of HIF1α/ARNT on the number of NIH3T3 cells. NIH3T3 cells were co-transfected with the Per2 promoter reporter (400 ng) and the indicated expression plasmids (300 ng) for HIF1α/ARNT or empty vector pcDNA3 (600 ng) as a control. Plates were read on the ArrayScan XTI (Thermo Scientific) for cell count indicated time after dexamethasone treatment. Numbers of viable cells were not affected by HIF1α/ARNT at all time points (mean ± SEM, *n* = 6, Student's *t*-test).

To determine whether HIF1α enhance the amplitude of oscillation of *Per2* promoter activities by increasing the number of cells, we performed cell count by using ArrayScan XTI (Thermo Scientific). Co-transfection with HIF1α/ARNT had no influence on the number of NIH3T3 cells (Fig. 4). This indicates that HIF1α/ARNT increased the bioluminescence of *Per2* promoter activities not affecting the number of cells.

### HIF1α directly binds to the HRE-like sequence within the *Per2* promoter

**Figure 5:**
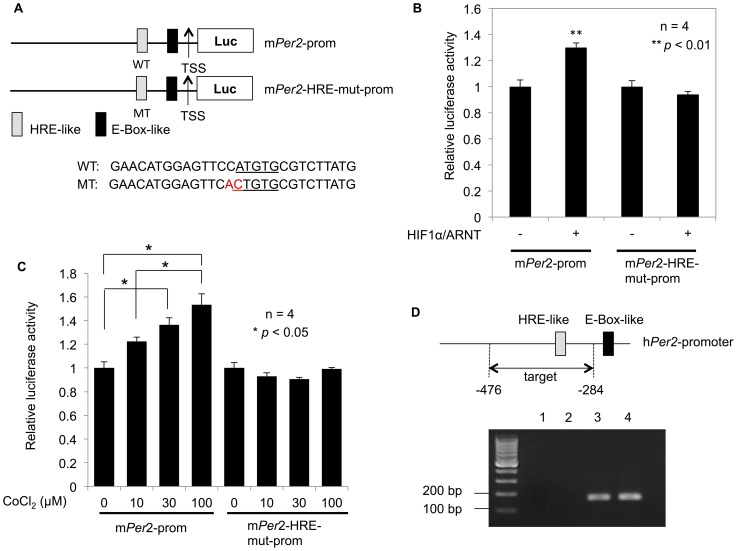
The effects of HIF1α/ARNT on the *Per2* promoter. (**A**) Schematic representation of the mouse *Per2* promoter. The upper area represents the wild-type mouse *Per2* promoter and the lower area represents the HRE-mutant *Per2* promoter. (**B**) HIF1α/ARNT potently induced *Per2* promoter activity. The *Per2* promoter and the HRE-mutant *Per2* promoter reporter (60 ng) were co-transfected with the indicated expression plasmids (+; 50 ng). *Per2* promoter activities were significantly increased (mean ± SEM, *n* = 4, *p*<0.01, Student's *t* test) compared to the control (without the expression plasmid), but the HRE-mutant *Per2* promoter was not affected. (**C**) Twenty-four hours after treatment with CoCl_2_ (10, 30, 100 µM for 6 h), luciferase activity was measured. *Per2* promoter activities were significantly increased (mean ± SEM, *n* = 4, *p*<0.05, one-way ANOVA followed by Tukey's post hoc tests) concentration-dependently compared to the control, but the HRE-mutant *Per2* promoter was not affected. (**D**) HIF1α specifically interacts with the HRE-like sequence within the *Per2* promoter. Caki-2 cells were cross-linked, lysed, and immunoprecipitated with anti-HIF1α antibody or normal rabbit IgG (negative control). The precipitated DNA was subjected to PCR with primers specific for the target region (−476/−284). One aliquot of input DNA was used as a positive control. PCR product was observed in the anti-HIF1α ChIP (lane 3) and 10% Input DNA (lane 4). Substantially less was detected in the no antibody ChIP (lane 1) and normal rabbit IgG ChIP (lane 2) lanes.

To determine whether HIF1α affected *Per2* transcription through the HRE-like element, a putative HIF1α-binding sequence, we examined the effects of HIF1α/ARNT on *Per2* expression using a luciferase assay in NIH3T3 cells. HIF1α/ARNT increased *Per2* transcriptional activity, but had no effect on HRE-mutant *Per2* promoters (Fig. 5A, B). CoCl_2_ treatment induces HIF1α expression by binding to the PAS domain, resulting in blockage of HIF1α-pVHL binding and thereby HIF1α stability [26], [27]. To investigate the effect of CoCl_2_-induced HIF1α overexpression on *Per2* transcriptional activity, cells were treated with CoCl_2_. CoCl_2_ upregulated *Per2* transcription but had no effect on the HRE-mutant *Per2* promoter (Fig. 5C). These results suggest that the HRE-like sequence in the *Per2* promoter we cloned responded to HIF1α overexpression. To investigate whether HIF1α directly binds to the HRE-like sequence in the *Per2* promoter *in vivo*, a ChIP assay was performed. Cross-linked Caki-2 cells were immunoprecipitated with rabbit anti-HIF1α antibody or normal rabbit IgG. The resulting immunoprecipitates were analyzed by PCR assays using primers flanking the HRE-like sequences (−476 to −284 bp) of the *Per2* promoter. A noticeable increase in the intensity of the DNA band was observed for the rabbit anti-HIF1α antibody (Fig. 5D, lane 3) but not for normal rabbit IgG (Fig. 5D, lane 2). These results indicated that HIF1α may increase *Per2* transcriptional activity by directly binding to the HRE-like element and enhance the amplitude of oscillation of *Per2* promoter activities.

### The effect of inhibiting HIF1α on the *Per2* circadian rhythm

**Figure 6:**
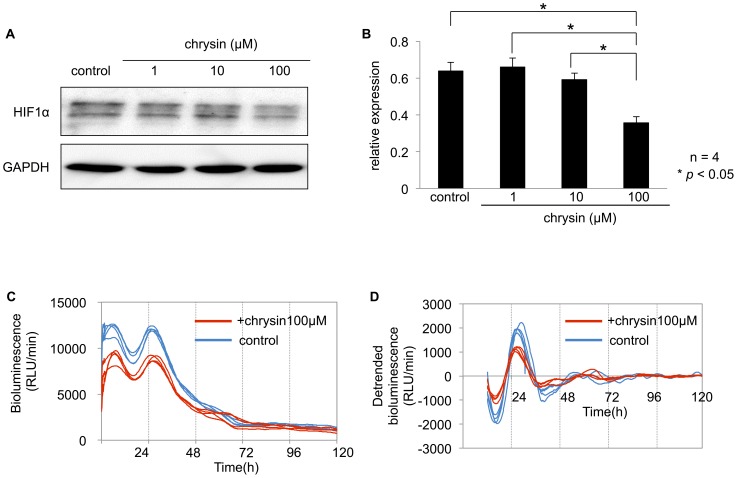
The effect of HIF1α inhibition on *Per2* circadian rhythm in Caki-2 cells. (**A**) Caki-2 cells were cultured to 60–70% confluence. The cells were treated with DMSO as a control, or different chrysin concentrations (1, 10, 100 µM) for 2 h. (**B**) HIF1α protein levels were measured in optical density values normalized to their respective GAPDH loading control, then averaged ± SEM, and graphed (relative expression) to semiquantitatively compare protein levels (*n* = 4). HIF1α expression was significantly suppressed by 100 nM chrysin compared to the control incubated with DMSO (*p*<0.05, one-way ANOVA followed by Tukey's post hoc test). (**C**) Caki-2 cells were transfected with the *Per2* promoter reporter (2 µg). Twenty-four hours after the transfection, cells were incubated with 100 µM chrysin or DMSO for 2 h. Bioluminescence was then measured using a real-time monitoring assay. The luciferase activities of four replicate samples are shown. (**D**) Detrended bioluminescence is shown. Period, amplitude, and acrophase of the oscillations were measured on days 2 to 5 using the Cosinor software (Circadian Rhythm Laboratory). Amplitude significantly decreased (mean ± SEM, *n* = 4) compared to the control (*p*<0.05). See Table 3.

Chrysin is a natural flavonoid, which is known to inhibit HIF1α expression by reducing protein synthesis and thereby decreases HIFα stability without affecting cell viability [28]. To examine the effect of inhibiting HIF1α protein on *Per2* circadian rhythm, the cells were pretreated with different chrysin concentrations. The expression of HIF1α protein was significantly suppressed after a 2-h incubation with 100 µM chrysin in Caki-2 cells (Fig. 6A, B). The amplitude of the circadian rhythm of the *Per2* promoter activity significantly decreased after a 2-h incubation with 100 µM chrysin in Caki-2 cells (Fig. 6C, D, Table 3). Based on these results, HIF1α may enhance the circadian rhythms of *Per2* at the promoter level.

**Table:** **Table 3.** Circadian parameters of *Per2* promoter activities based on four days of data in Caki-2 cells.

	period (h)	amplitude	acrophase (h)	P value
control	24.3±0.04	1045.57±41.99	5.49±0.10	<0.000001
+chrysin100 µM	24.2±0.04	766.08±23.95*	5.74±0.11	<0.000001

Period, amplitude, and acrophase of the oscillations were measured on days 2 to 5 using the Cosinor software (Circadian Rhythm Laboratory). Amplitude significantly decreased (mean ± SEM, *n* = 4) compared to the control (* *p*<0.05).

## Discussion

In this study, rhythmic expression of the *Per2* gene was observed in Caki-2 cells. However, *Per2* promoter activities and mRNA levels did not have circadian rhythms in any other cell lines. Some differences may exist between Caki-2 and other renal cancer cell lines. Because *Per2* gene transcription is activated by the heterodimerized transcription factor BMAL1/CLOCK by binding to the E-box-like sequence [24], we examined the expression of BMAL1 and CLOCK protein in these cell lines. Caki-2, 786-O, and A498 cells expressed BMAL1, and all cell lines contained CLOCK protein. Furthermore, all renal cancer cell lines expressed CRY1 protein, but did not express PER2 protein. In Caki-2 cells, we also examined the expression of PER2 protein at 4-h intervals beginning 24 h after treatment with dexamethasone. However, no PER2 protein was detected at all time points (Fig. S4). No definitive mechanism to account for the discrepancy between the lack of PER2 protein expression and positive mRNA expression in Caki-2 cells has yet been elucidated. It is reported that the protein kinase CK2 specifically binds and phosphorylates PER2 and interacts with the protein kinase CKIε to promote PER2 degradation [29]. Furthermore, the expression levels and activities of CK2 are reported to be increased in many tumors and tumor cell lines [30]. Thus, in this case, there is a possibility that posttranslational modification by CK2 causes the loss of PER2 protein. However, further studies will be needed to elucidate the detailed mechanism underlying CK2-mediated enhancement of CKIε-dependent PER2 degradation.

Upregulation of HIF1α may be common in RCC since *VHL* is often mutated [31]. Thus, we examined the expression of HIF1α protein in these cell lines. We found that Caki-2 and RCC4+vector alone cells contained HIF1α protein. Taken together, rhythmic expression of the *Per2* gene was observed in Caki-2 containing BMAL1, CLOCK, and HIF1α protein. This suggested that both BMAL1/CLOCK and HIF1α may be related to the circadian expression of *Per2* in renal cancer cell lines. Thus, we hypothesized that HIF1α may affect circadian expression of the *Per2* gene. In the present study, HIF1α increased the amplitude of the *Per2* circadian rhythm in Caki-2 cells, as well as mouse and human models. Sequence analysis of the *Per2* promoter region revealed high homology between mice and humans. These results suggest that the promoter region we cloned is suitable for use in experiments on human cell lines. We also examined the difference between the wild-type m*Per2* promoter and the HRE-mutant m*Per2* promoter in NIH3T3 cells without HIF1α or CoCl_2_. The transcriptional activity and amplitude of the HRE-mutant m*Per2* promoter was higher than those of the wild-type m*Per2* promoter (Fig. S5 A–C). It is possible that the factor which has negative effects on the HRE-like sequence can not bind to the mutated HRE-like sequence, hence the transcriptional activity and amplitude of the HRE-mutant m*Per2* promoter was higher than those of the wild-type m*Per2* promoter. Moreover, another factor might upregulate the *Per2* transcriptional activity and amplitude of oscillation by binding to the mutated HRE-like sequence. We are investigating these factors, however the detailed mechanism remains unclear. This will be addressed in future studies. Based on our luciferase assay with HIF1α and CoCl_2_, the HRE-like element we identified may respond to overexpression of HIF1α. Furthermore, ChIP assay revealed the direct binding of HIF1α to the HRE-like element. These results indicate that HIF1α may activate *Per2* transcription and increase the amplitude of the *Per2* circadian rhythm by directly binding to the HRE-like element within the *Per2* promoter.

As shown in Fig. 6, inhibition of HIF1α decreased the amplitude of the circadian rhythm of the *Per2* promoter activities. These data support that HIF1α plays a role in the circadian expression of *Per2* in renal cancer cell lines.

We hypothesized that *Per2* circadian rhythms in Caki-2 cells were produced by BMAL1/CLOCK and HIF1α following our observation that HIF1α may enhance the *Per2* circadian rhythms. However, further investigation is required to determine the molecular mechanisms of HIF1α-induced enhancement of *Per2* circadian rhythms and differences between Caki-2 and other renal cancer cell lines.

Rhythmic expression of the *Per2* gene was not observed in renal cancer cell lines, excluding Caki-2 cells. Although rhythmicity of *Per2* promoter activities was determined using online software, bioluminescence levels in Caki-2 cells were much lower than in NIH3T3 and U2OS cells. Rhythmic expression of the *Bmal1* gene was not observed in any renal cancer cell line (Fig. S6). These results suggest that disruption of circadian rhythms of clock genes may be common in renal cancer cell lines. Previous studies revealed that circadian rhythm disruption in mice is associated with accelerated growth of malignant tumors [32]. Also, *Per2* plays an important role in setting the period of oscillation [14] and has tumor-suppressor properties [33]. The *Per2* gene functions in tumor suppression by regulating DNA-damage-responsive pathways, and *Per2*-deficient mice show signs of premature aging and increased neoplastic tissue development following gamma irradiation [34]. Mutations in the *Per2* gene have been identified in human colorectal and breast cancers^17^. Moreover, overexpression of *Per2* inhibits tumor proliferation in culture and in animals [35], [36]. In the present study, HIF1α may activate *Per2* transcription and increase the amplitude of the *Per2* circadian rhythm by directly binding to the *Per2* promoter. Considering that *Per2* is reported to have tumor-suppressor properties, it is possible that HIF1α increases *Per2* transcriptional activity thereby inhibits tumor proliferation in contrast to the previous finding that HIF-mediated gene pathway is the main risk-factor of tumor growth in renal cancer. However, a recent study showed that, in contrast to previous reports, deficiency in either the *Per1* or *Per2* gene alone does not render mice more tumor-prone; moreover, some long-term effects of ionizing radiation in *Per2*-deficient mice are more reminiscent of accelerated aging rather than a tumor-prone phenotype [37]. Therefore, we should investigate the detailed molecular mechanisms of *Per2* as potentially important targets for renal cancer therapy and clarify the effect of HIF induced *Per2* upregulation on tumor progression in renal cancer in future study. Furthermore, their detailed role that seems to conflict with the role of HIF-mediated angiogenic pathway should be investigated as well.

## Supporting Information

Figure S1 Real-time monitoring of luciferase activity of the *Per2* promoter in renal cancer cell lines and NIH3T3 cells. Rhythmicity was not detectable in renal cancer cell lines (*p*>0.05, by Cosinor). The amount of plasmid and Lipofectamine 2000 used in each of the cell lines are shown.
(TIFF)

Figure S2 Full-length blots of BMAL1. (**A**) Full-length blots of BMAL1 in indicated cell lines including a circadian competent cell line (NIH3T3) as a positive control are presented. (**B**) The positive controls using a circadian competent cell line (NIH3T3) transfected with the indicated expression plasmids are presented.
(TIFF)

Figure S3 Full-length blots of PER2. The positive controls using a circadian competent cell line (NIH3T3) and HeLa cells, which is listed in manufacturer's datasheet as a positive control, are presented.
(TIFF)

Figure S4 Full-length blots of PER2 in Caki-2 cells at seven time points.
(TIFF)

Figure S5 Difference between the wild-type m*Per2* promoter and the HRE-mutant m*Per2* promoter in NIH3T3 cells without HIF1α or CoCl_2_. (**A**) Difference between the relative luciferase activities of the wild-type m*Per2* promoter and the HRE-mutant m*Per2* promoter in NIH3T3 cells. (**B**) Bioluminescence of the wild-type m*Per2* promoter and the HRE-mutant m*Per2* promoter in NIH3T3 cells. Four replicate samples are shown. (**C**) Detrended bioluminescence of the wild-type m*Per2* promoter and the HRE-mutant m*Per2* promoter in NIH3T3 cells. Four replicate samples are shown.
(TIFF)

Figure S6 Real-time monitoring of luciferase activity of the *Bmal1* promoter in renal cancer cell lines and NIH3T3 cells. Rhythmicity was not detectable in renal cancer cell lines (*p*>0.05, by Cosinor). The amount of plasmid and Lipofectamine 2000 used in each of the cell lines are shown.
(TIFF)
