# Supplementary figures and images for: The Impact of HIF1α on the Per2 Circadian Rhythm in Renal Cancer Cell Lines
Source: PLoS One. 2014 Oct 21;9(10):e109693. doi: 10.1371/journal.pone.0109693 (PMC4204850; doi:10.1371/journal.pone.0109693)

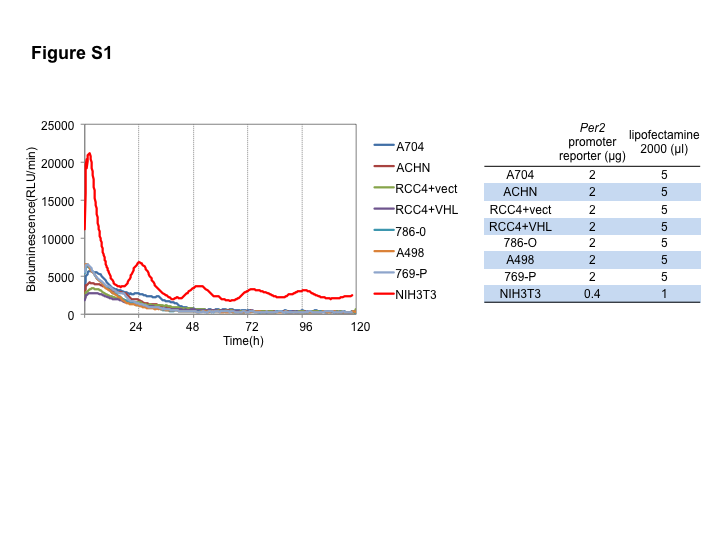

Supplement: Figure S1 — Real-time monitoring of luciferase activity of the Per2 promoter in renal cancer cell lines and NIH3T3 cells. Rhythmicity was not detectable in renal cancer cell lines (p>0.05, by Cosinor). The amount of plasmid and Lipofectamine 2000 used in each of the cell lines are shown. (TIFF) [file pone.0109693.s001.tiff]

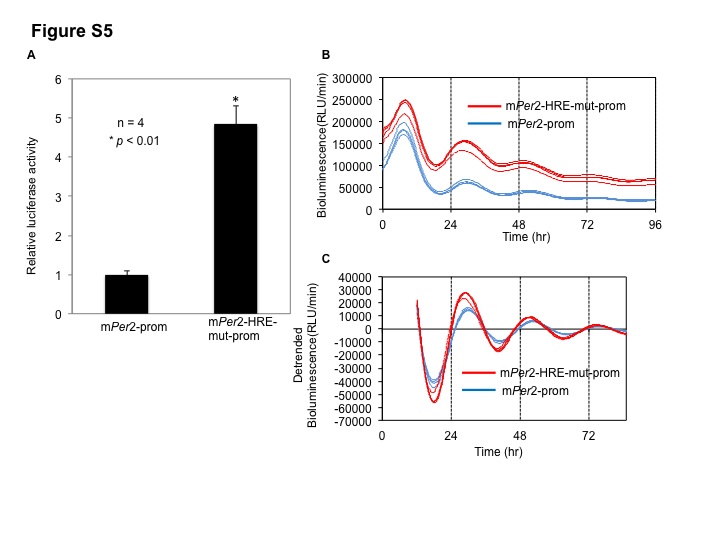

Supplement: Figure S5 — Difference between the wild-type mPer2 promoter and the HRE-mutant mPer2 promoter in NIH3T3 cells without HIF1α or CoCl2. (A) Difference between the relative luciferase activities of the wild-type mPer2 promoter and the HRE-mutant mPer2 promoter in NIH3T3 cells. (B) Bioluminescence of the wild-type mPer2 promoter and the HRE-mutant mPer2 promoter in NIH3T3 cells. Four replicate samples are shown. (C) Detrended bioluminescence of the wild-type mPer2 promoter and the HRE-mutant mPer2 promoter in NIH3T3 cells. Four replicate samples are shown. (TIFF) [file pone.0109693.s005.tiff]

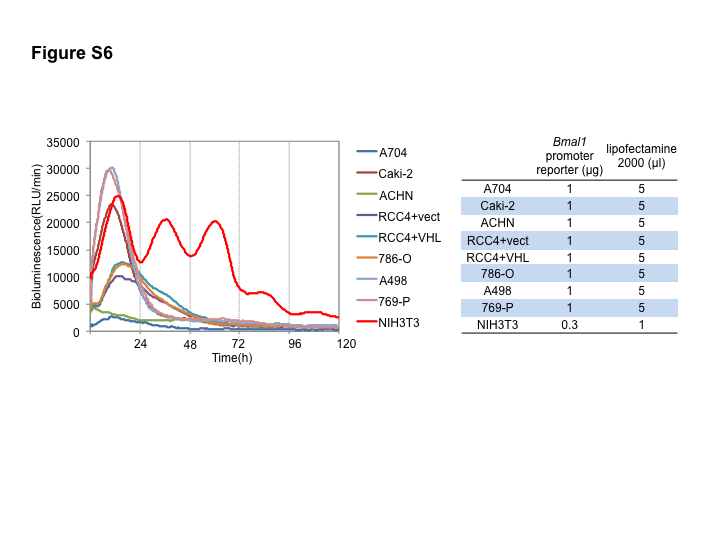

Supplement: Figure S6 — Real-time monitoring of luciferase activity of the Bmal1 promoter in renal cancer cell lines and NIH3T3 cells. Rhythmicity was not detectable in renal cancer cell lines (p>0.05, by Cosinor). The amount of plasmid and Lipofectamine 2000 used in each of the cell lines are shown. (TIFF) [file pone.0109693.s006.tiff]
